# The *Fasciola hepatica* genome: gene duplication and polymorphism reveals adaptation to the host environment and the capacity for rapid evolution

**DOI:** 10.1186/s13059-015-0632-2

**Published:** 2015-04-03

**Authors:** Krystyna Cwiklinski, John Pius Dalton, Philippe J Dufresne, James La Course, Diana JL Williams, Jane Hodgkinson, Steve Paterson

**Affiliations:** Institute of Infection and Global Health, University of Liverpool, Liverpool, UK; School of Biological Sciences, Medical Biology Centre, Queen’s University of Belfast, Belfast, Northern Ireland UK; Institute of Parasitology, McGill University, Montreal, Quebec Canada; Institut National de Santé Publique du Québec, Montreal, Quebec Canada; Liverpool School of Tropical Medicine, Liverpool, UK; Institute of Integrative Biology, University of Liverpool, Liverpool, UK

## Abstract

**Background:**

The liver fluke *Fasciola hepatica* is a major pathogen of livestock worldwide, causing huge economic losses to agriculture, as well as 2.4 million human infections annually.

**Results:**

Here we provide a draft genome for *F. hepatica*, which we find to be among the largest known pathogen genomes at 1.3 Gb. This size cannot be explained by genome duplication or expansion of a single repeat element, and remains a paradox given the burden it may impose on egg production necessary to transmit infection. Despite the potential for inbreeding by facultative self-fertilisation, substantial levels of polymorphism were found, which highlights the evolutionary potential for rapid adaptation to changes in host availability, climate change or to drug or vaccine interventions. Non-synonymous polymorphisms were elevated in genes shared with parasitic taxa, which may be particularly relevant for the ability of the parasite to adapt to a broad range of definitive mammalian and intermediate molluscan hosts. Large-scale transcriptional changes, particularly within expanded protease and tubulin families, were found as the parasite migrated from the gut, across the peritoneum and through the liver to mature in the bile ducts. We identify novel members of anti-oxidant and detoxification pathways and defined their differential expression through infection, which may explain the stage-specific efficacy of different anthelmintic drugs.

**Conclusions:**

The genome analysis described here provides new insights into the evolution of this important pathogen, its adaptation to the host environment and external selection pressures. This analysis also provides a platform for research into novel drugs and vaccines.

**Electronic supplementary material:**

The online version of this article (doi:10.1186/s13059-015-0632-2) contains supplementary material, which is available to authorized users.

## Background

The digenean trematode *Fasciola hepatica* is one of the most important pathogens of domestic livestock and has a global distribution [1-4]. The disease, fasciolosis, results in huge losses to the agricultural industry associated with poor food conversion, lower weight gains, impaired fertility and reduced milk (cattle) and wool (sheep) production. Heavy, acute infections can result in death, particularly in sheep and goats. Economic losses attributable to *F. hepatica* infection have been estimated at more than US$3 billion per annum worldwide [5,6], although even this estimate may be conservative as *F. hepatica* infection modulates its host’s immune response and its ability to resist or eliminate common microbial pathogens [7,8]. Fasciolosis is also an important zoonosis in regions where agricultural management practices are less advanced, particularly in South America and North Africa [3,9]. It is estimated that between 2.4 and 17 million people are infected with this liver fluke worldwide, with a further 91 million people living at risk, resulting in fasciolosis being included on the World Health Organization list of major neglected tropical diseases [1-3].

**Figure 1:**
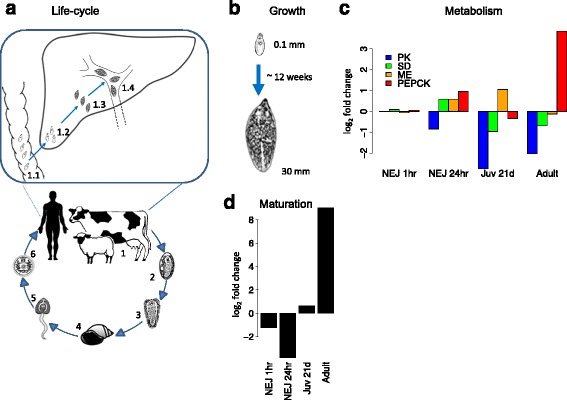
*Fasciola hepatica* lifecycle. **(a)** Graphical representation of the *F. hepatica* lifecycle (modified from [76]). *a1* Definitive host - host range includes cattle, sheep and humans. *a1.1* Parasite excysts in the intestine of the definitive host, releasing newly excysted juveniles (NEJ) that migrate across the intestinal wall, through the peritoneal cavity to the liver. *a1.2* NEJ migrate through the liver parenchyma, increasing in size to juvenile flukes as they migrate *a1.3* into the bile ducts *a1.4*, where they grow and develop into fully mature adults. *a2* Eggs are released in the faeces and develop on pasture. *a3* From each embryonated egg hatches a single miracidium, which infects the snail intermediate host (*Galba truncatula*). *a4* Within the snail the parasite undergoes a clonal expansion, developing through the sporocyst, rediae and cercariae lifecycle stages. *a5* Cercariae are released from the snail and encyst on vegetation as dormant metacercariae, which are ingested by the definitive host *a1*. **(b, c, d)** Graphical representation of the development of the parasite through the definitive host. **(b)** The parasite increases dramatically (approximately 1,000-fold) in size over the course of approximately 12 weeks, from NEJ to adult. **(c)** Expression of key enzymes of metabolism reveals how the growth of the parasite limits oxygen diffusion into the parasite tissue, switching from aerobic energy metabolism (Kreb’s cycle; PK: pyruvate kinase; SD: succinate dehydrogenase) to aerobic acetate production (ME: malic enzyme) to anaerobic dismutation (PEPCK: phosphoenolpyruvate carboxykinase), as shown by the log fold-change in expression between the lifecycle stages (expression is shown relative to metacercariae lifecycle stage). **(d)** In addition to the dramatic growth, maturation of the parasite occurs, with the fully mature adult digesting host blood, which provides the nutrient for massive egg production (approximately 20,000 eggs per day per parasite), as shown by the increased expression of the egg shell component, vitelline.

The zoonotic potential of *F. hepatica* is enabled by its remarkable ability to infect and mature in an extensive range of terrestrial mammals. Thus, while the typical definitive host for *F. hepatica* is one of many species of domestic or wild ruminant that ingest contaminated pasture (Figure 1), *F. hepatica* is also able to exploit disparate host species including humans and rodents, and has rapidly adapted to novel hosts such as llamas and kangaroos, which it has recently come into contact with in South America and Australia, respectively [10]. This is in contrast to most digenean trematodes, such as the human pathogen *Schistosoma mansoni*, which have a far more restricted host range. *F. hepatica* can also adapt rapidly to drug interventions and the emergence of resistance within *F. hepatica* populations to triclabendazole (TCBZ) is of major concern, since most drugs used against other digeneans are only partly protective against *F. hepatica* [10]. TCBZ is also the only drug currently available that is able to protect livestock and humans against early stage juveniles, which cause significant pathology as they migrate through the liver. The ability of *F. hepatica* to adapt rapidly to novel hosts or to drug interventions is perhaps more remarkable given that *F. hepatica* is a hermaphrodite that can facultatively self-fertilise and so *F. hepatica* populations might be expected to lose genetic diversity through inbreeding that would be an essential basis for adaptation.

Here we provide a genome assembly for *F. hepatica* and assess genome-wide polymorphism and transcriptional profiles in order to identify key features of its genome that underlie its ability to migrate through different physiological environments, to parasitise different host species, and to respond rapidly to external selection pressures.

## Results and discussion

### A large genome with high gene polymorphism

A draft genome for *F. hepatica* was generated with an assembled length of approximately 1.3 Gb (Table 1). The genome of *F. hepatica* is considerably larger than that of other sequenced digenean parasites - *Schistosoma spp* (363 to 397 Mb), *Clonorchis sinensis* (547 Mb) or *Opisthorchis viverrini* (634.5 Mb) [11-16] - and is one of the largest pathogen genomes sequenced to date. Genome size does not appear to be related to chromosome number among trematodes; *F. hepatica* has 10 pairs of chromosomes [17], *S. mansoni* and *O. viverrini* have eight pairs and six pairs of chromosomes, respectively [18,19], but *C. sinensis*, also with a smaller genome than *F. hepatica*, has 28 pairs [20]. Comparative analysis with other sequenced trematode species indicated that the mean number of exons per gene is comparable between species, but that mean exon and intron lengths tend to increase with genome size (Additional file 1: Table S1). Most core eukaryotic genes appeared as single copy evidenced by both CEGMA (Additional file 2: Table S2) and analysis of read coverage (Additional file 3: Figure S1), suggesting that the large genome size of *F. hepatica* has not arisen by genome duplication. At least 32% of the genome was estimated to consist of repetitive DNA, which is consistent with other trematode genomes [11-15]. The median repeat length was 26 bp (Additional file 4: Figure S2) and we observed retrotransposons, including 27 Mbp of long terminal repeats and 59 Mbp of long interspersed elements (LINEs); however, there was no obvious expansion of a single repeat element to account for the large genome size. A LINE RTE BovB repeat, previously found in ruminants, was observed distributed widely across the genome (at least 67,000 full or partial copies in total across approximately 30% of the scaffolds and totalling 28.1 Mbp). This was not due to contamination by host DNA, since no other host sequence, such as sheep mitochondrial sequence, could be identified either in the assembly or in individual reads. BovB has previously been reported as exhibiting horizontal transfer between snakes and ruminants and its presence in *F. hepatica* suggests that transfer of BovB elements between disparate vertebrate taxa may be facilitated by digenean infection [21].

**Table 1:** ***Fasciola hepatica*** **assembly statistics**

**Metric**	**Value**
Scaffold N50	204 Kbp (REAPR 155 Kbp^a^)
Number of scaffolds ≥3 Kbp	20,158
Number of scaffolds ≥1 Kbp	45,354
Contig N50 (≥100 bp)	9.7 K bp
Number of contigs (≥100 bp)	254,014
Total assembly length	1.275 Gbp
Total length of gaps	91.6 Mbp
Repetitive content	32%
Number of RNAseq-supported gene models	22,676 (15,740^b^)
Mean number of exons/gene	5.3
Mean exon size (95% range)	303 bp (36 bp – 1,369 bp)
Mean intron size (95% range)	3.7 Kbp (33 bp - 17.5 Kbp)
Proportion CEGMA core eukaryotic genes found	90%

^a^N50 following breakage of some scaffolds at areas to low support. Both assemblies are available from ENA under project accession PRJEB6687.

^b^Number of non-overlapping, distinct genome intervals covered by RNAseq-supported gene models.

We investigated levels of polymorphism among *F. hepatica* genes by re-sequencing the genomes of individual fluke from each of five isolates, all from the UK. Substantial polymorphism among isolates was observed; 48% of genes exhibited at least one non-synonymous SNP and the level of non-synonymous nucleotide diversity, pi, averaged across 21.8 Mbp of coding sequence, was 5.2 × 10^-4^ (that is, two randomly sampled sequences differed approximately every 1,900 bp). By comparison, this figure is higher than in humans [22], similar to most vertebrates [23] and, on limited data, smaller than some parasitic nematode populations [24]. Although *F. hepatica* is a self-fertilising hermaphrodite, and so has the potential to inbreed and lose genetic diversity, our data show that *F. hepatica* populations, as a whole, harbour substantial genetic variation. A likely explanation is that parasite populations are typically large, often larger than that of their hosts, which greatly slows any enhanced effects of genetic drift caused by self-fertilisation [25].

**Figure 2:**
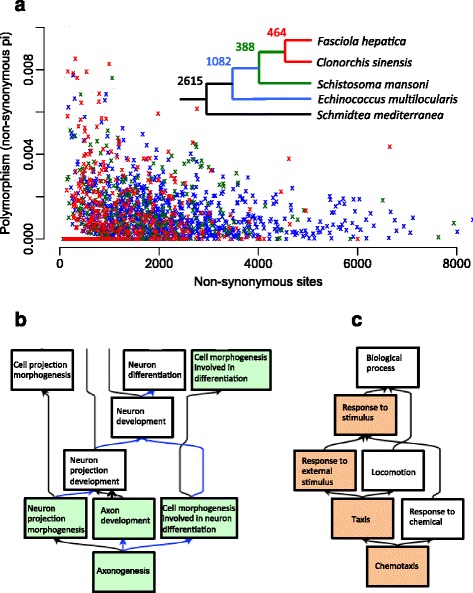
Polymorphism within *Fasciola hepatica*. **(a)** Levels of non-synonymous polymorphism for *F. hepatica* genes exhibiting orthology with *Clonorchis*, *Schistosoma*, *Schmidtea* or *Echinococcus* indicated within the phylogenic tree. Numbers by branches refer to numbers of orthologous groups specifically shared by that branch; for example, 464 orthologs are shared only between *Fasciola* and *Clonorchis*, a further 388 are also shared with *Schistosoma* but not with *Schmidtea* or *Echinococcus* and so on. Branches not drawn to scale. Polymorphism is significantly (*P* <0.001) higher in *Fasciola* orthologs shared among the digenean and cestode parasites *Clonorchis*, *Schistosoma* and *Echinococcus* (green, red and blue crosses, respectively) relative to orthologs conserved with the turbellarian *Schmidtea* (black dots). **(b, c)** Directed acyclic graphs indicating over-representation of biological processes within the top 1% most polymorphic genes, based on non-synonymous diversity. Shaded boxes indicated significant (*P* <0.01) over-representation. Black and blue arrows indicate, respectively, ‘is a’ and ‘part of’ relationships between terms.

By analysing the distribution of genetic diversity amongst *F. hepatica* genes, we found higher non-synonymous polymorphism in genes shared with parasitic cestodes and digeneans relative to orthologs shared with the free-living turbellaria (Figure 2a and Additional file 5: Table S3 and Additional file 6: Table S4). These data suggest high adaptability in *F. hepatica* genes that mediate infection and survival in the host environment, which is consistent with *F. hepatica*’s ability to infect a range of both mammalian and molluscan hosts [3,9,10]. We then assessed whether high non-synonymous polymorphism was associated with particular biological functions and discovered a marked over-representation of biological processes associated with axonogenesis and chemotaxis among the top 1% quantile of polymorphic genes (Figure 2b and Additional file 7: Table S5 and Additional file 8: Table S6). These genes included cadherin, semaphorin, fascilin and rabconnectin, which are involved in cell adhesion and migration of neurons [26-28]. The high polymorphism observed in chemosensory and neural development pathways may relate to the challenge faced by *F. hepatica* in locating its snail host or in tissue migration in its vertebrate host, and with variation in host preference within parasite populations [29,30]. Such polymorphism may be particularly relevant for development of new anthelmintics targeting the parasite’s neuromuscular system [31].

### Expression patterns from multi-gene families reveal important developmental host-parasite interactions

**Figure 3:**
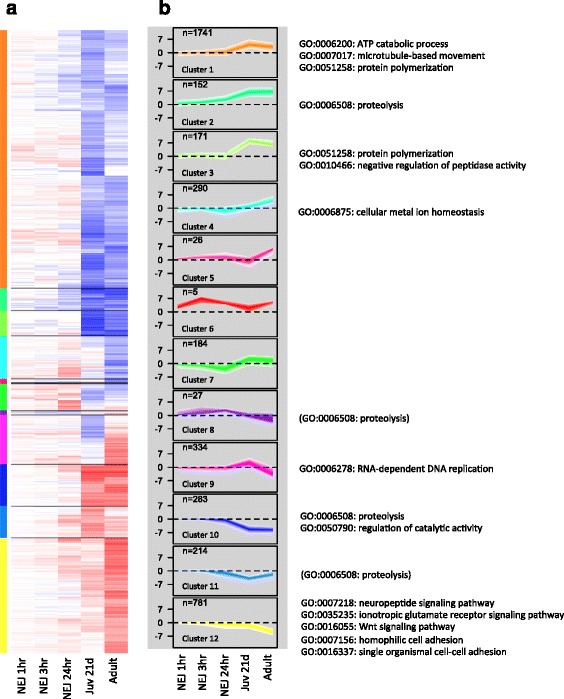
Expression of genes exhibiting at least a 16-fold difference in expression between any two developmental stages and grouped by hierarchical clustering. **(a)** Heatmap with upregulation in blue and downregulation in red relative to metacercariae. Colours to left of heatmap correspond to different clusters. **(b)** Expression of genes within each cluster on a log_2_ scale. The number of genes and the enrichment of biological processes are shown for the most specific terms within the gene ontology structure and with a significance of <0.1%. GO:0006508 Proteolysis is shown in brackets for clusters where it appears with a significance of 0.1% < *P* < 1%.

In order to understand how *F. hepatica* has adapted to survive within its vertebrate host, we characterised its developmental time-course of gene expression using RNAseq. Progressively more genes were differentially expressed, and with larger fold-changes, following initial infection and subsequent development in the host; that is, associated with excysting of the metacercaria stage to form the newly excysted juvenile (NEJ), traversal of the intestinal wall and penetration of the liver as an immature tissue-migrating parasite (Figure 3a). Thereafter, the transition from these immature parasites to mature adults living in the bile duct was accompanied by further changes in the overall profile of differentially expressed genes, but not in the number of genes differentially expressed relative to metacercariae. Known aspects of *Fasciola* biology were recapitulated in our expression data. Thus, as the parasite grows, diffusion of oxygen into parasite tissue is limiting and our expression analysis confirmed the switch from aerobic to anaerobic metabolism (Figure 1b and c) [32]. Similarly, our data confirmed that the production of vitelline, the egg shell component, is limited to the mature parasites (Figure 1d).

To explore novel processes associated with developmental changes, genes were clustered based on expression pattern (Figure 3b). Several biological processes were greatly downregulated in maturing and adult parasites relative to metacercariae (Cluster 12, Figure 3), including molecules involved in cell adhesion (cadherins, integrins) and cytoskeletal proteins (talins) that could play an important role in sensing changes in the physiological environment and rapidly initiating excystment following ingestion by the definitive host. Clusters showing strongest patterns of differential expression (both up- and downregulation) were markedly over-represented by peptidases and terms associated with regulation of peptidase activity (Clusters 2, 8, 10 and 11, Figure 3). Genes associated with *F. hepatica* structure, particularly protein polymerisation and microtubule based movement, such as tubulin, dynein and surface tegumental genes, were highly upregulated in immature and adult fluke relative to earlier stages (Clusters 1 and 3, Figure 3b and Additional file 9: Table S7, Additional file 10: Table S8 and Additional file 11: Table S9). Our data show that, across the transcriptome as a whole, the strongest changes in expression were observed among different members of the multigenic protease and tubulin families, as detailed below.

**Figure 4:**
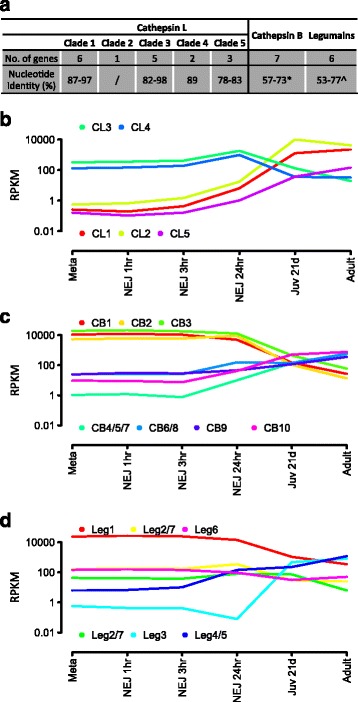
Analysis of the cysteine proteases belonging to *F. hepatica* cathepsin L (FhCL) and cathepsin B (FhCB) clades and their activators, the asparaginyl endopeptidases/legumains. **(a)** Representation of the number of genes identified for each FhCL clade and for the groups of FhCB and legumain genes, based on analysis by BLAST and manual annotation. The nucleotide identity is shown for each clade/group of genes (Clustal Omega). **(b, c, d)** Graphical representation of the expression for FhCL, FhCB and legumain proteases across the *F. hepatica* lifecycle in reads per kilobase per million (RPKM). In the case of the FhCL gene expression, all the genes within the same clade showed a similar pattern of expression, and so are represented here as an average log RPKM. Likewise in the case where multiple gene models were identified for a particular gene, which showed a similar level of expression, are represented here as an average log RPKM.

Peptidases play essential roles in parasite infection, tissue migration and feeding [33]. *F. hepatica* is unique among helminth parasites because it relies almost exclusively on the secretion of papain-like cysteine peptidases (Clan A, family C1), classes cathepsins L (FhCL) and cathepsins B (FhCB) [33,34]. These two peptidase classes, however, have expanded and diverged to form multigenic families, all of which have were found within our assembly (Figure 4a and Additional file 12: Figure S3). Our data show that FhCL peptidases form clades with a total of 17 members and we found that each clade has a distinctive peptidolytic activity based on residues that make up the S2 sub-site of the active site (Additional file 12: Figure S3 [35,36]). Members within each clade exhibited a concerted pattern of expression that can be correlated with migration of the parasite through different tissues where they would encounter a different profile of macromolecular substrates. For example, FhCL3s are known to exhibit a unique collagenolytic activity required to disrupt the interstitial matrix and their secretion in high amounts enable NEJs to traverse the intestinal wall and penetrate the liver capsule [33,34,37]. Our data reveal that the FhCL3 clade has expanded to five members that are all abundantly expressed by NEJs and share critical residues (H61, W67 and V205) within the active site, suggesting that enlargement of this family has been driven by the need to produce plentiful collagenolytic peptidase at this critical time in the parasite’s life cycle. Conversely, members of the FhCL1 clade, the largest with six members, were not expressed during the early migratory stages but all showed an increase in expression as the parasite matures (Figure 4b). Mature adults are obligate blood feeders [30,37] and secrete copious amounts of FhCL1 and, correspondingly, evolution of the FhCL1 active site (residues N61, L/F67 and L/M205) has resulted in loss of collagenolytic activity but a gain in the ability to effectively digest blood proteins, particularly haemoglobin, which are the mature parasite’s prime source of nutrients required for egg production [30,37].

Unlike the FhCL peptidases, the FhCB family consists of a single clade of seven members. Nevertheless, these peptidases were also temporally regulated (Figure 4c) such that three members (FhCB1, FhCB2 and FhCB3) exhibited parallel expression patterns to FhCL3 and were thus highly expressed in the NEJs and downregulated as the parasites matured. These data suggest a concerted role for FhCLs and FhCBs in the early infection stage. Also, the constitution and specific expression of a family of peptidases, asparaginyl endopeptidases or legumains that are responsible for the processing of the inactive FhCL and FhCB zymogens to functional enzymes [38] suggest specific and important developmental roles for these peptidases (Figure 4d). Thus, legumain 1, which was the most highly expressed member in NEJs could be required for activation of the FhCL3 and FhCB 1/2/3 peptides at the time the parasite emerges from its encysted stage in the intestine and initiates infection. Furthermore, legumain 3, which was switched on late in parasite development, is the prospective candidate for activating the FhCL1, FhCL2 and FhCL5 in mature blood-feeding adult parasites.

**Figure 5:**
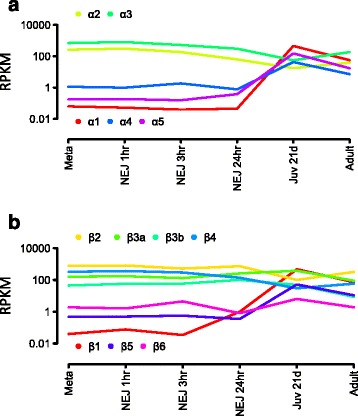
Expression of tubulin genes. **(a, b)** Graphical representation of the expression of the tubulin genes across the *F. hepatica* lifecycle represented by α-tubulins **(a)** and β-tubulins **(b)**. The expression of each tubulin isotype (iso) is shown in reads per kilobase per million (RPKM), which were calculated as an average log RPKM value for the multiple transcriptome datasets at each lifecycle stage.

Our data highlight tubulins as another multigenic family that exhibit among the highest log fold-changes in expression throughout *F. hepatica* development. We identified the full complement of five α-tubulin and six β-tubulin isotypes [39,40] and found duplication of β-tubulin isotype 3 (Figure 5a, b). Basal expression levels revealed two functional subsets of β-tubulin molecules; high constitutive expression of β-tubulin isotypes 2, 3a, 3b and 4 suggests they play an essential role in general microtubule structure and function, while a more specialised role is implicated for β-tubulin isotype 1 and 5, which were particularly expressed in the immature liver stages. Tubulins are known targets of benzimidazole (BZ) drugs [40,41], which include TCBZ, and the unique nature of the interaction of *F. hepatica* with TCBZ remains undefined. Protein similarity searches with the closely related species *C. sinensis* and *S. mansoni*, which are refractory to TCBZ, identified six and 10 β-tubulin gene models, respectively. While *F. hepatica* β-tubulin isotypes 1 to 4 show >95% similarity with multiple sequences from these species, that observed for β-tubulin isotype 5 and 6 was <70% (Additional file 13: Table S10). Of the two BZ-derived drugs effective against *F. hepatica*, TCBZ targets immature and adult fluke while albendazole (ABZ), a widely used anti-nematode drug, kills only adult stages. Field isolates of *F. hepatica* that demonstrate resistance to ABZ remain susceptible to TCBZ suggesting independent modes of action [42] that may be related to the diverse β-tubulin isotypes found in this parasite.

### Anti-oxidant and detoxification systems

We investigated the evolution of anti-oxidant systems in *F. hepatica*, which are essential for adaptation to the host environment. Thus, as the parasite rapidly develops and enters different aerobic/anaerobic environments, anti-oxidant systems are critical not only for the detoxification of reactive oxygen and nitrogen (ROS, RNS) generated by endogenous cellular metabolism but also as a frontline defence against superoxide and nitric oxide radicals released by host immune effector cells such as macrophages, eosinophils and neutrophils [43,44]. Parasitic platyhelminths express genes encoding superoxide dismutase (SOD) which dismutates superoxide radicals to H_2_O_2_ but they do not possess catalase, the enzyme responsible for converting H_2_O_2_ to water and oxygen (although a catalase gene is present in the genome of free-living flatworms, such as *Schmidtea mediterranea*). A catalase gene was not found in the *F. hepatica* genome, which is consistent with other parasitic platyhelminths, where the function of peroxide detoxification has been supplanted by the newly discovered thiol-dependent peroxiredoxin and its reducing partner thioredoxin [45]. The presence of the gene encoding the recently described thioredoxin glutathione reductase (TGR; Additional file 14: Table S11), together with the absence of distinct thioredoxin reductase and glutathione reductase genes verified that TGR is the sole reductive enzyme that links the thioredoxin-dependent and glutathione-dependent anti-oxidant defence systems in this parasite as observed in other platyhelminths [43,46,47]. The pivotal position of TGR between these two essential anti-oxidant systems makes it a promising target for the development of new anti-trematode drugs [46]. Included in the repertoire of *F. hepatica* anti-oxidants are genes encoding SOD, glutathione transferases (GSTs) and three fatty acid binding proteins (FABP; Additional file 14: Table S11). With the exception of the GSTs, we found that in *F. hepatica* each of these components is encoded by a single gene, which is in stark contrast to the expanded anti-oxidant gene families in the closely related trematodes, *O. viverrini* and *C. sinensis* [13,15]. Peroxiredoxin, GST and FABP are secreted by *F. hepatica* via non-classical secretory pathways, perhaps as cargoes of exosomes [37], into the host circulation where they influence host immune responses by recruiting and activating M2 macrophages [7,48,49] and suppressing dendritic cell activity [50]. These immunomodulatory effects contribute to the establishment of an immune suppressive environment, which aids parasite survival and the development of chronic disease.

**Figure 6:**
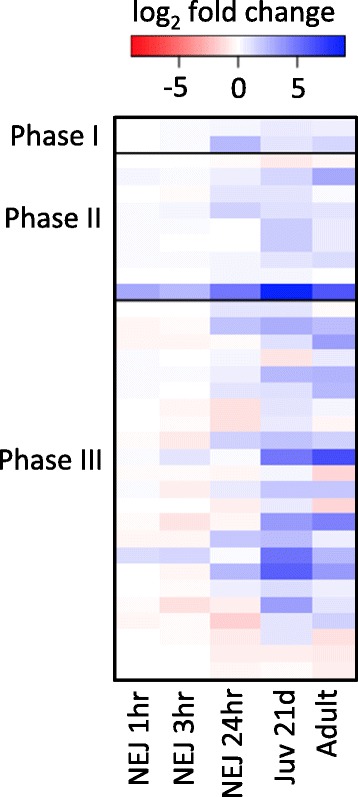
Expression of detoxification genes. Expression of Phase I, II and III detoxification genes through development relative to metacaercariae.

Defence against chemical toxins, including those generated by the immune response, is essential in allowing helminths to adapt to, and survive in, the host environment. This is mediated in large part by the three-phase drug detoxification pathway; Phase I (activation), Phase II (conjugation) and Phase III (efflux). This pathway also acts to reduce drug activity and/or bioavailability and has been linked with TCBZ resistance [41,51-53]. Until now only indirect evidence existed for the presence of Phase I cytochrome P450 (CYP 450) genes in *F. hepatica* [54]. Here, we report that the *F. hepatica* genome contains two Phase I CYP 450 gene models, one monooxygenase and one reductase; both are present in *S. mansoni* but only the reductase is found in *C. sinensis*. Basal expression levels did not reveal significant changes throughout development (Figure 6). Our analysis reveals that of the Phase II GSTs, the cytosolic GSTs were represented in the *F. hepatica* genome by at least seven separate genes comprising four different classes; four mu, one omega, one sigma and a putative novel zeta class. Zeta class GSTs demonstrate different activities to other GSTs and their role in helminths is poorly understood [55]. Comparison with *S. mansoni* and *C. sinensis* revealed this zeta class GST is unique to *F. hepatica*. Within the Phase III pathway [56,57], we identified 18 ATP-Binding Cassette (ABC) and five Multidrug and Toxic compound Extrusion (MATE) transporters in *F. hepatica*, similar to that found in *S. mansoni* and *C. sinensis*. Although no significant developmental changes in gene expression were observed for the cytosolic GSTs, we found that two microsomal GSTs and several putative multidrug resistance proteins were upregulated in the immature and adult stages (Figure 6 and Additional file 15: Table S12) supporting the importance of broad-spectrum detoxification Phases in mediating defence against host-immune-mediated toxic assault and targeting via anti-parasitic drugs.

## Conclusions

The *F. hepatica* genome is one of the largest pathogen genomes sequenced to date but we found no evidence of genome duplication or repeat expansion to explain this. Why this large genome should have evolved is unclear, especially given that its replication may be energetically costly or slow cell division [58]. For a parasite, such as *Fasciola*, that relies on the production of large numbers of eggs to facilitate transmission, one would expect strong selection against the accumulation of junk DNA if a large genome imposed a cost on egg production. It is possible that much of the non-coding portion of the *F. hepatica* genome is involved in gene regulation [59], and the size of *F. hepatica*’s genome may be related to its complex life cycle and variety of developmental morphs. If so, however, it would be difficult to explain why the *F. hepatica* genome is around three time the size of the *Schistosoma* genome [11], which has a similar life cycle. The large genome of *F. hepatica* therefore remains a paradox for which we may have to wait for comparative genome sequencing, currently underway [60], across other platyhelminth taxa to provide an answer.

The ability of *F. hepatica* to infect and survive in different tissue environments as it migrates from the intestine, through the liver and into the bile ducts is underpinned by gene duplication. Thus, our results show that, across the whole transcriptome, the strongest patterns of differential expression were observed among members of protease and tubulin gene families, and, in the case of proteases, these can be associated with changes in the active site and substrate specificity. While gene duplication appears to be a key process of adaptation to the parasitic life-style used by many helminths, it is notable that different helminth taxa have arrived at different evolutionary solutions. Comparison between *F. hepatica* and the bile-dwelling liver flukes *C. sinensis* and *O. viverrini*, shows the expansion of the anti-oxidant SOD families [13] and cathepsin F protease families [13,61] in *C. sinensis* and *O. viverrini* but not in *F. hepatica,* and conversely the expansion of the cathepsin L family in the latter species only, which suggests differences in how these related parasites tackle life within the same environment. Differences in the specificity or developmental expression of detoxification genes, such as members of the ABC and MATE families, or of the tubulin gene family, may be important in understanding why *F. hepatica* responds so markedly different to drugs at different stages of development compared to other digeneans. The exclusive activity of TCBZ against *F. hepatica*, and the potency of praziquantel to all these other digeneans except *F. hepatica*, points to a uniqueness in this parasite that needs to be resolved [37].

*Fasciola hepatica* is a highly adaptable parasite, evidenced by its ability to infect novel hosts, and it is notable that our results reveal high levels of polymorphism in genes specific to parasitic digeneans. Diversity within *F. hepatica* populations at genes important for the host-parasite interface may underpin a high evolutionary potential for *F. hepatica* to respond to changes in host availability or to other selection pressures. Similarly, the broad geographic range of *F. hepatica* would suggest that it is able to adapt to different climatic conditions and, in this respect, *F. hepatica* may also be able to respond to exploit changes in climate in temperate regions, such as the UK, where warmer and wetter winters favour transmission and increased prevalence [62]. *F. hepatica* is seen to rapidly develop drug resistance to TCBZ [52] and the standing genetic diversity that we find across the genome suggests that it harbours the potential to evolve resistance to any novel drug treatment, which compounds the difficultly of controlling *F. hepatica* given the shortage of drugs effective against the juvenile stages. Nevertheless, the availability of a genome for *F. hepatica* that we provide, plus the characterisation of early molecular events in infection should help support the development of novel drugs and vaccines, particularly against the migrating juveniles that cause much of the pathology. For example, RNAi-mediated knockdown of cathepsin L and B expression has been shown to prevent NEJs from crossing the intestine [45] and vaccine trials with cathepsin L1 have provided partial protection against infection and pathology [63]. The exploitation of a broader range of targets within the *F. hepatica* genome is now a priority given the widespread prevalence of resistance to TCBZ within *F. hepatica* populations. The commercial importance for agriculture to develop a replacement treatment to TCBZ may stimulate new treatments that could be translated to other important digenean parasites, including those of humans.

## Methods

### Source of parasite material

Adult parasites from each of five isolates were used for genome sequencing: (1) FhepLivSP, from the laboratory maintained Shrewsbury isolate (Ridgeway Research, UK); (2) FhepLivS1, a clonal line derived from the Shrewsbury population; (3) and (4) FhepLivR1 and R3, clonal lines derived from two isolates recovered from sheep in Northern England naturally infected with *F. hepatica*; and (5) FhepLivR2, a clonal line derived from a *F. hepatica* population from naturally infected sheep in South West Wales. For RNA sequencing, the following were used: (1) metacercariae and newly excysted juveniles (NEJ) at 1, 3 and 24 h post excystment from a North American isolate (Baldwin Aquatics Inc., Monmouth, OR, USA). Twenty-one–day-old juvenile flukes were recovered from mice infected with the same isolate; (2) an adult parasite recovered from the bile ducts of cattle naturally infected with *F. hepatica* in Uruguay. All animal work was conducted with ethical approval from the Universities of Liverpool (UK) and McGill (Canada).

### Sequencing

Approximately 10 μg of DNA from a single, adult fluke taken from the FhepLivS1 strain was used to prepare Illumina TruSeq fragment libraries and 26 Gbp of 2 × 250 bp reads generated on an Illumina MiSeq (mean insert sizes 470 bp and 580 bp). Further individuals of FhepLivS1 were used to prepare Nextera Mate-Pair libraries (3 Kbp and 10 Kbp) and approximately 60 m 2× 100 bp Illumina reads from each library were generated. Approximately 200 μg of DNA prepared from several fluke of the FhepLivSP isolate was used to construct 2 Kbp, 5 Kbp and 8 Kbp mate-pair libraries, which were sequenced either on an Illumina GAII or HiSeq 2000. For each of the parasite isolates FhepLivSP, FhepLivS1, FhepLivR1, FhepLivR2 and FhepLivR3, DNA was isolated from a single adult; a fragment library was prepared and sequenced on a single lane of an Illumina HiSeq 2000 to yield approximately 24 Gbp of sequence (Centre for Genomic Research, Liverpool, UK). For RNA sequencing, Illumina TruSeq RNA libraries were prepared from biological replicates of metacercariae (3 replicates), NEJ 1 h (2 replicates), NEJ 3 h (2 replicates), NEJ 24 h (2 replicates), Juveniles 21 days (1 replicate) and Adult (1 replicate) (Genome Quebec, Montreal, Canada).

### Assembly and annotation

Illumina MiSeq reads were trimmed to Q ≥30 and adaptors removed using Sickle and Perl and assembled using Newbler (Roche GS-Assembler v2.6) with flags set for large genome and a heterozygote sample. Mate-pair reads were first mapped to these contigs using Bowtie2 [64] to remove duplicates and wrongly orientated reads, and scaffolded into contigs using SSPACE [65]. Gap filling was achieved using GapFiller for 2× 250 bp and 2× 100 bp paired-end reads and run for three iterations (available as ENA accessions LN627018-LN647175). RNAseq data were mapped to scaffolds within the assembled genome greater than 3 Kbp using TopHat2 to identify transcribed regions and splice junctions. These, together with RNAseq data assembled using Trinity and *S. mansoni* protein sequence, were passed to the MAKER pipeline [66] to predict genes. Repeatmasker, Windowmasker and Dustmasker were used to identify repetitive regions. CEGMA v2.4 [67], which searches for 248 highly conserved genes, was used to assess the completeness of genome with the settings for vertebrates to allow long introns. REAPR [68] was used to assess the quality of scaffolding within the assembly and to produce an alternative, more conservative assembly by splitting scaffolds at locations with lower support (available as ENA accessions LN736597-LN774150). Homologs of *F. hepatica* predicted protein sequences were identified within UniProt using BLAST and functional domains identified using InterPro. InParanoid and MultiParanoid [69] were used to identify ortholog clusters from *Schistosoma mansoni* (v3.1.16), *Clonorchis sinensis* (v3.7), *Schmidtea mediterranea* and *Echinococcus multilocularis* (v29042013) predicted proteins [11-15,70].

### Gene expression analysis

RNAseq libraries were mapped to MAKER gene models using TopHat2 [71] and read counts extracted using htseq-count. Genes with a sum of at least five reads across all libraries were analysed for differential expression in edgeR [72] using a negative binomial model of successive developmental stages relative to metacercariae and with tagwise dispersion estimated from all samples. Hierarchical clustering, based on model coefficients, was used to group differentially expressed genes by similarity of expression into 12 clusters and hypergeometric tests used to test for over-representation of gene ontology terms within each cluster relative to the whole gene-set.

### Genetic diversity analysis

Reads for each isolate were mapped to the genome using Bowtie2 and resulting bam files passed together to the GATK pipeline [73] for local realignment and SNP calling. SNP calls within genes were filtered by score >100, FS <60 and combined coverage between 10 and 250. Because gene duplicates collapsed within the assembly might give erroneously high diversity for some genes, genes were excluded with a median coverage greater than 213 and by heuristic scoring of SNPs appearing as heterozygotes in all samples. IGV was used to manually assess the success of filtering parameters. Levels of nucleotide diversity for different classes of SNPs were calculated for all genes. To identify the most polymorphic genes, a generalised linear model with a Poisson distribution was fitted to the number of non-synonymous SNPs as the response variable versus number of non-synonymous sites as a quadratic function. This accounts for the distribution of SNP counts within a gene, the fact that longer genes have the potential to have more SNPs and the possibility that genes of different lengths may evolve at different rates. Genes were classified according to their conservation across platyhelminths by the presence of orthologs retained across increasing taxonomic scales. This was preferred to assessing conservation on the basis of non-synonymous versus synonymous substitution ratios between species, since synonymous sites were saturated by multiple substitutions at such broad taxonomic scales. The robustness of the generalised linear model was tested by randomly sampling equal proportions of genes from each quartile of the length distribution for each class of conserved gene, to ensure that no bias was introduced if the length of a gene was correlated to its level of conservation. From the generalised linear model, the most polymorphic genes were identified having residuals in the top 1% quantile and hypergeometric tests were used to test for over-representation of GO terms within these highly polymorphic genes relative to the gene-set as a whole.

### Discovery and characterisation of gene families

Discovery of *F. hepatica* gene families was carried out using BLAST analysis (NCBI v2.2.27 and v2.2.29), with available published gene sequences of interest (Additional file 16: Table S13), followed by manual annotation. Comparative analysis was carried out using the closely related trematode genome sequence datasets: *Clonorchis sinensis* and *Schistosoma mansoni*. Sequence alignment and phylogenetic analysis was carried out using Clustal Omega [74] and MEGA5 [75], respectively.

### Data availability

Data are freely available from WormBase ParaSite and the European Nucleotide Archive under accessions LN627018-LN647175 (assembly data), PRJEB6687 (genomic read data) and PRJEB6904 (transcriptomic read data).

## Additional files

Additional file 1: Table S1. Comparison of the *F. hepatica* genome with other parasitic trematode genomes. Additional file 2: Table S2. Summary statistics of CEGMA analysis. Additional file 3: Figure S1. Distribution of average read coverage across gene models. Additional file 4: Figure S2. Distribution of repeat length within the *Fasciola hepatica* genome. Additional file 5: Table S3. Number of *Fasciola hepatica* ortholog clusters identified in *Clonorchis sinensis*, *Schistosoma mansoni*, *Schmidtea mediterranea* and *Echinococcus multilocularis*. Additional file 6: Table S4. Association between number of non-synonymous SNPs per gene and orthology with other platyhelminths. Showing the output of a generalised linear model with a quasi-poisson error structure. Additional file 7: Table S5. Over-representation of gene ontology (GO) terms in the top 1% quantile polymorphic genes, as estimated from number of non-synonymous SNPs controlled for length. Additional file 8: Table S6. Top 1% most polymorphic genes. Additional file 9: Table S7. Over-respresentation of GO Terms associated with each co-expression cluster. Additional file 10: Table S8. Identity of gene models within each of 12 co-expression clusters, including annotation. Additional file 11: Table S9. Differential expression of genes presented in Figure 3. Values are log_2_ fold-changes relative to metacercaria. Additional file 12: Figure S3. Analysis of the clades representing the cathepsin L cysteine proteases. Additional file 13: Table S10. Comparative analysis of the *F. hepatica* β tubulin genes with *C. sinensis* and *S. mansoni*, based on protein similarity. Additional file 14: Table S11.
*F. hepatica* antioxidant analysis. Additional file 15: Table S12. Differential expression of detoxification genes. Additional file 16: Table S13. Table of *Fasciola* sequences used for BLAST analysis.
