# Supplementary material for: The Fasciola hepatica genome: gene duplication and polymorphism reveals adaptation to the host environment and the capacity for rapid evolution
Source: Genome Biol. 2015 Apr 3;16(1):71. doi: 10.1186/s13059-015-0632-2 (PMC4404566; doi:10.1186/s13059-015-0632-2)
Supplement: Additional file 3: Figure S1. — Distribution of average read coverage across gene models. [file 13059_2015_632_MOESM3_ESM.pdf]

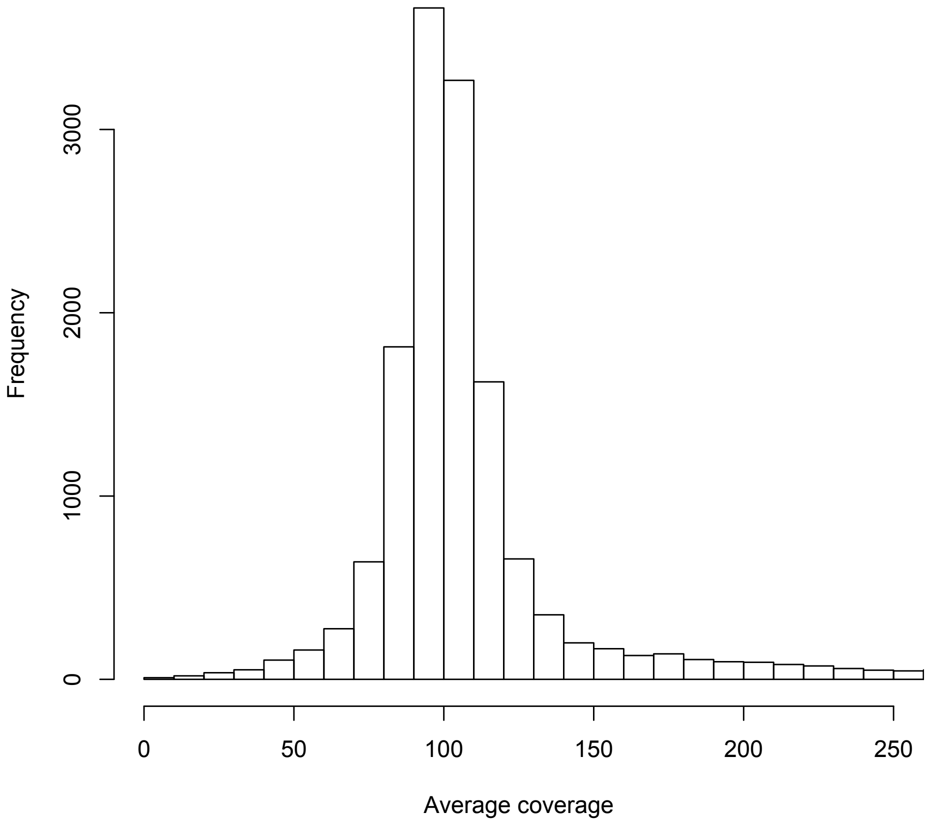

Figure S1. Distribution of average read coverage across gene models.
Data from resequencing of all 5 *Fasciola hepatica* isolates was used to generate an average coverage for each gene and the frequency distribution of average coverage across all genes generated as a histogram. The single peak observed indicates that the assembly does not contain either a significant proportion of alleles that have failed to assemble into a single locus, or of paralogous genes erroneously assembledinto a single locus.
