# Supplementary figures and images for: The Fasciola hepatica genome: gene duplication and polymorphism reveals adaptation to the host environment and the capacity for rapid evolution
Source: Genome Biol. 2015 Apr 3;16(1):71. doi: 10.1186/s13059-015-0632-2 (PMC4404566; doi:10.1186/s13059-015-0632-2)

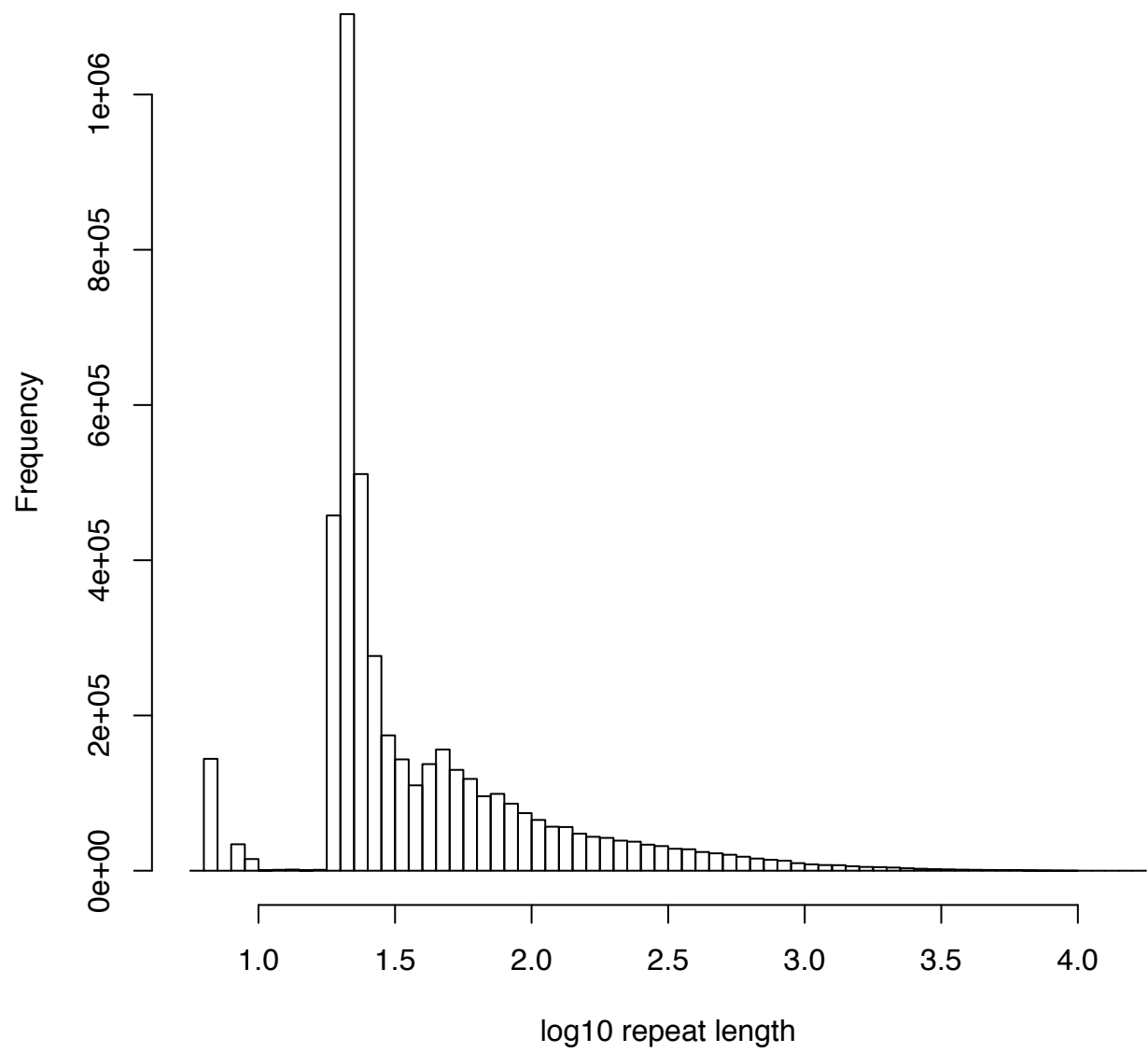

**Figure S2.** Distribution of repeat length within the *Fasciola hepatica* genome.

Supplement: Additional file 4: Figure S2. — Distribution of repeat length within the Fasciola hepatica genome. [file 13059_2015_632_MOESM4_ESM.pdf]
