# Supplementary material for: The Fasciola hepatica genome: gene duplication and polymorphism reveals adaptation to the host environment and the capacity for rapid evolution
Source: Genome Biol. 2015 Apr 3;16(1):71. doi: 10.1186/s13059-015-0632-2 (PMC4404566; doi:10.1186/s13059-015-0632-2)
Supplement: Additional file 12: Figure S3. — Analysis of the clades representing the cathepsin L cysteine proteases. [file 13059_2015_632_MOESM12_ESM.pdf]

A

```
CL1         MRLFILAVLTVGVLGSNDDLWHQWKRMYNKEYNGADDQHRRNIWEENVKHIQEHNLRHDL 60
CL4         MRLFILAILTFEVFASNDDLWHEWKRMYNKEYNGVDDAHRRNIWEENVKHIQEHNIRHDL 60
CL5         MRLIILTLLAVGVFASNDDLWHQWKQTYNRKHHGADDEKRRNIWEQNVKHIQEHNLRHDL 60
CL2         MRFFVLAVLTVGVFASNDDLWHQWKRIYNKEYNGADDEHRRNIWGKNVKHIQEHNLRHDL 60
CL3         MRLLILAVLFAGAFASNDVSWHEWKRMYNKEYNGADDEHRRNIWEQNVKHIEEHNLRHDR 60

CL1         GLVTYTLGLNQFTDMTFEEFKAKYLTEMSRASDILSHGVPYEANNRAVPDKIDWRESGYV 120
CL4         GLVTYTLGLNQFTDMTFEEFKATYLREIPRASDMLSHGIPYEAKDRAAPVSIDWREFGYV 120
CL5         GLVTYRLGLNQFTDMTFEEFKAKYLSKMPRASELLSHGMPYRAKNRAVPASIDWRESGYV 120
CL2         GLVTYKLGLNQFTDLTFEEFKAKYLIEIPRSSELLSRGIPYKANKLAVPESIDWRDYYYV 120
CL3         GLVTYKLGLNQFTDLTFEEFKAKYLMEMSPVSESLSDGISYEAEGNDVPASIDWRQYGYV 120

                                                                     *
CL1         TEVKDQGNCGSCWAFSTTGTMEGQYMKNERTSISFSEQQLVDCSGPWGNNGCSGGLMENA 180
CL4         TEVKDQGKCGSCWAFSTTGAVEGQYMKNQKTNISFSEQQLVDCSGDYGNNGCSGGLMENA 180
CL5         TEVKDQGGCGSCWAFSTTGAMEGQYMKSQRINISFSEQQLVDCSGDFGNHGCSGGLMEKA 180
CL2         TEVKNQGQCGSCWAFSTTGAVEGQFRKNERASASFSEQQLVDCTRDFGNYGCGGGYMENA 180
CL3         TEVKDQGQCGSCWAFSAVGAIEGQYVKKFQNQTLFSEQQLVDCTRRFGNHGCGGGWMENA 180

CL1         YQYLKQFGLETESSYPYTAVEGQCRYNKQLGVAKVTGYYTVHSGSEVELKNLVGAEGPAA 240
CL4         YEYLWEHGLETESSYPYKAVEGPCKYDIRLGVAKVTGYYLVHSGIESVLQDLVGAEGPAA 240
CL5         YEYLRHFGLETESSYSYRADEGPCQYDRQLGVAQVSGYYIVHSQDEVALKNLIGVEGPAA 240
CL2         YEYLKHNGLETESYYPYQAVEGPCQYDGRLAYAKVTGYYTVHSGDEIELKNLVGTEGPAA 240
CL3         YKYLKNSGLETASYYPYQGWEYQCQYRKELGVAKVTGAYTVHSGDEMKLMQMVGREGPAA 240

                                              *
CL1         VAVDVESDFMMYRSGIYQSQTCSPLRVNHAVLAVGYGTQGGTDYWIVKNSWGLSWGERGY 300
CL4         VGVDAELDFMLYKSGIYESRNCSSESLNHGILVVGYGTQDGTDYWIVKNSWGSLWGEHGY 300
CL5         VALDVNIDFMMYRSGIYQDEICSSRYLNHAVLAVGYGTEDGTDYWIVKNSWGPLWGEHGY 300
CL2         VALDADSDFMMYQSGIYQSQTCLPDRLTHAVLAVGYGSQDGTDYWIVKNSWGTWWGEDGY 300
CL3         VAVDAQSDFYMYESGIFQSQYCSSRRVTHAVLAVGYGTESGTDYWILKNSWGKWWGEDGY 300

                            *
CL1         IRMARNRGNMCGIASLASLPMVARFP 326
CL4         IRMARNRDNMCGIASLASLPVVEPFP 326
CL5         IRLARNRDNMCGIATLASLPIVKRFP 326
CL2         IRFARNRGNMCGIASLASVPMVARFP 326
CL3         MRFARNRGNMCAIASVASVPMVERFP 326
```

B

Clade consensus sequence	Residue position											
	17	23	61	67	68	133	157	158	159	160	178	205
CL1	Q	C	N	L	M	A	L	N	H	A	N	L
CL5	Q	C	H	L	M	A	L	N	H	A	N	-
CL4	Q	C	-	-	M	A	L	N	H	G	N	L
CL2	Q	C	Y	Y	M	A	L	T	H	A	N	L
CL3	Q	C	H	W	M	A	V	T	H	A	N	V

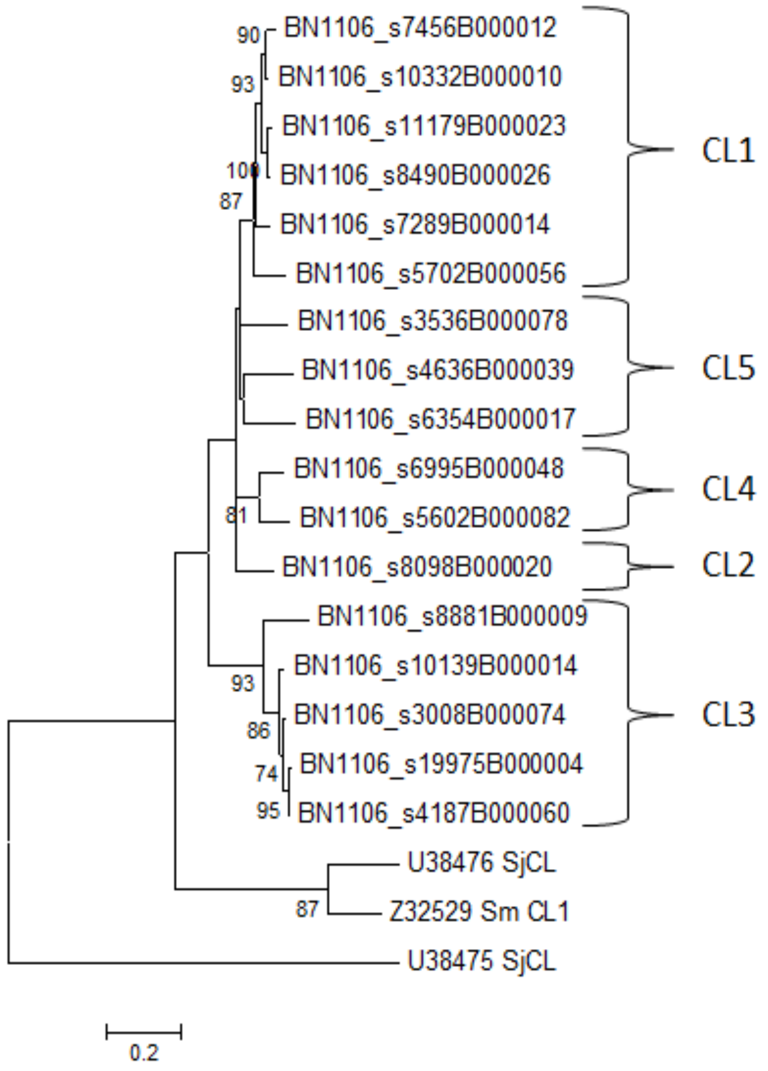

C

Figure S3. Analysis of the clades representing the cathepsin L cysteine proteases. (a) Protein alignment using representative sequences for each clade of cathepsin L protease. The genomic organisation of these genes is conserved across all the cathepsin L genes identified within the *F. hepatica* genome, regardless of which clade the proteases belong to. Intron-exon borders, resulting in four exons are indicated by the black arrows. The signal peptide and pro-segment domain are highlighted in green and blue, respectively, with the short blue arrow indicating the position of the pro-protein cleavage site. The residues in bold and shaded in grey represent the active site residues, that comprise the S1 binding subsite. The residues highlighted in red represent those residues that comprise the S2 binding subsite. The particular S2 residues that confer substrate specificity are highlighted by the red *. (b) Comparison of the residues from the S1 and S2 binding subsites across the cathepsin L clades. Variability across the sequences represented by the different genes within each clade is shown. (c) Phylogenetic analysis of the *F. hepatica* cathepsin L gene family, based on the genes identified within the *F. hepatica* genome. A maximum likelihood tree was constructed with the nucleotide sequence corresponding to the prosegment of the protein including the catalytic domain. For three genes the sequences are represented by two gene models (*). The tree is drawn to scale and branch lengths are measured in number of substitutions per site using MEGA v 5.05 (Tamura et al., 2011). Bootstrap values >70% from 100 iterations are shown. The tree is rooted using cathepsin L sequences from the closely related trematode species, *Schistosoma mansoni* and *Schistosoma japonicum* (SjCL: U38476; SmCL1; Z32529; SjCL: U38475).
